# Molecular technique reveals high variability of 18S rDNA distribution in harvestmen (Opiliones, Phalangiidae) from South Africa

**DOI:** 10.3897/CompCytogen.v12i1.21744

**Published:** 2018-02-13

**Authors:** František Šťáhlavský, Vera Opatova, Pavel Just, Leon N. Lotz, Charles R. Haddad

**Affiliations:** 1 Department of Zoology, Faculty of Science, Charles University, Viničná 7, CZ-12844 Praha, Czech Republic; 2 Department of Biological Sciences and Auburn University Museum of Natural History, Auburn University, Auburn, AL 36849, USA; 3 Department of Arachnology, National Museum, P.O. Box 266, Bloemfontein 9300, South Africa; 4 Department of Zoology and Entomology, University of the Free State, P.O. Box 339, Bloemfontein 9300, South Africa

**Keywords:** Karyotype, meiosis, sex chromosomes, FISH, 18S rDNA

## Abstract

The knowledge of cytogenetics in the harvestmen family Phalangiidae has been based on taxa from the Northern Hemisphere. We performed cytogenetic analysis on *Guruia africana* (Karsch, 1878) (2n=24) and four species of the genus *Rhampsinitus* Simon, 1879 (2n=24, 26, 34) from South Africa. Fluorescence *in situ* hybridization with an 18S rDNA probe was used to analyze the number and the distribution of this cluster in the family Phalangiidae for the first time. The results support the cytogenetic characteristics typical for the majority of harvestmen taxa, i.e. the predominance of small biarmed chromosomes and the absence of morphologically well-differentiated sex chromosomes as an ancestral state. We identified the number of 18S rDNA sites ranging from two in *R. qachasneki* Kauri, 1962 to seven in one population of *R. leighi* Pocock, 1903. Moreover, we found differences in the number and localization of 18S rDNA sites in *R. leighi* between populations from two localities and between sexes of *R. capensis* (Loman, 1898). The heterozygous states of the 18S rDNA sites in these species may indicate the presence of XX/XY and ZZ/ZW sex chromosomes, and the possible existence of these systems in harvestmen is discussed. The variability of the 18S rDNA sites indicates intensive chromosomal changes during the differentiation of the karyotypes, which is in contrast to the usual uniformity in chromosomal morphology known from harvestmen so far.

## Introduction

The harvestmen (Opiliones) represent one of the oldest terrestrial arachnid lineages (Dunlop et al. 2004). They are traditionally divided into four suborders (Cyphophthalmi, Eupnoi, Dyspnoi and Laniatores) (e.g. Pinto-da-Rocha et al. 2007), altogether comprising more than 6500 described species (Kury 2017). Harvestmen are present on all continents (except Antarctica) (Giribet and Kury 2007) and have a wide range of ecological functions within the ecosystem (e.g. predators, detritivores, scavengers). Typically, harvestmen have limited dispersal capabilities and show tendencies to local endemicity (Giribet and Kury 2007). Despite the species richness and ancient geographical isolation of many groups, harvestmen possess similar cytogenetic characteristics within each suborder.

So far, the chromosomes of 90 species of harvestmen have been examined, which ranks them as the third best cytogenetically explored arachnid order (Tsurusaki et al. 2017). All groups usually have biarmed monocentric chromosomes (Tsurusaki 2007). The highest numbers of chromosomes are known from Laniatores (2n=25–109, median 81) (Schneider et al. 2008) and Cyphopthalmi (2n=24–52, median 30) (Svojanovská et al. 2016). Both Dyspnoi (2n=10–28, median 16) and Eupnoi (2n=10–36, median 22) typically possess a lower number of chromosomes (Tsurusaki et al. 2017). In some species, intraspecific variability in chromosome numbers has been reported. Given the low vagility of harvestmen, the variability could be caused by a fixation of local karyotype changes in isolated populations. Geographical variation among populations is particularly known from sabaconids (Dypnoi) and sclerosomatids (Eupnoi) (Tsurusaki 2007), where narrow hybrid zones exist between the populations with different chromosome races (e.g. Tsurusaki et al. 1991, Gorlov and Tsurusaki 2000a). However, the intraspecific variability in the chromosome number may also be attributed to the presence of B chromosomes, i.e. the supernumerary chromosomes that remain univalent during meiosis and are usually heterochromatic and non-coding. The number of B chromosomes can vary even within one individual (White 1973). In opilionids, the presence of B chromosomes has been documented in the sclerosomatid harvestman *Psathyropus tenuipes* L. Koch, 1878 (Eupnoi), where some individuals possessed up to 18 B chromosomes (Tsurusaki 1993). Finally, the intraspecific variability could also be related to the presence of the heteromorphic XX/XY or ZZ/ZW sex chromosome systems. The XX/XY system evolved independently in both Dyspnoi and Eupnoi (Tsurusaki 2007), while the ZZ/ZW system is documented uniquely in *Mitopus morio* (Fabricius, 1779) (Eupnoi: Phalangiidae) from Rishiri island in Japan (Tsurusaki and Cokendolpher 1990).

The majority of the available chromosome data on harvestmen originates from the suborder Eupnoi (58 species examined) (Tsurusaki et al. 2017). However, all the Eupnoi taxa examined so far, with exception of *Holmbergiana weyenberghii* (Holmberg, 1876) (Sclerosomatidae) from Argentina (Rodríguez Gil and Mola 2010), occur in the Northern Hemisphere. Despite our relatively good knowledge of the cytogenetics of this group, it is likely that the variability within Eupnoi could still be underestimated, due to the obvious geographical bias of the available data. The suborder Cyphophthalmi could serve as an example of a similar situation; it was traditionally perceived as cytogenetically uniform, but a surprising amount of diversity was revealed when analyses encompassed material from a broad geographical range (Svojanovská et al. 2016).

The suborder Eupnoi is divided into six families comprising 1822 species (Pinto-da-Rocha et al. 2007, Kury 2013). The family Phalangiidae (394 described species), with the centre of diversity in the Northern Hemisphere (Giribet and Kury 2007), represents the second most diverse group of Eupnoi (Kury 2017). The presence of Eupnoi in South Africa is traditionally explained by the group’s active dispersion from the Mediterranean region (Staręga 1984). Presently, only one species, *Guruia africana* (Karsch, 1878), and 30 species of the genus *Rhampsinitus* Simon, 1879 belonging to the family Phalangiidae are known from South Africa (Lotz 2009). In this study, we analyse the karyotypes of South African representatives of the family Phalangiidae and present the first data on the cytogenetics of this family of harvestmen from the Afrotropical Region. Moreover, we also use fluorescence in situ hybridization (FISH) to identify the number and position of the 18S rRNA genes, which is the first implementation of this method in the family Phalangiidae. The marker for 18S rRNA is frequently used to reveal concealed karyotype differences in groups with similar chromosomes (e.g. Cabral de Mello et al. 2011, Mattos et al. 2014, Sember et al. 2015) and to help inferring specific chromosomal changes along the course of the karyotype evolution (e.g. Nguyen et al. 2010, Grzywacz et al. 2011, Panzera et al. 2012).

## Material and methods

The material examined in this study is deposited in the National Museum, Bloemfontein, South Africa (NMBA). We analyzed five species of harvestmen belonging to the family Phalangiidae from different localities in South Africa:

*Guruia africana* (Karsch, 1878): KwaZulu-Natal: Ndumo Game Reserve (26.8749°S, 32.2109°E), 4 males (NMBAO00900–NMBAO00903).

*Rhampsinitus capensis* (Loman, 1898): Eastern Cape: Hogsback (32.5888°S, 26.9352°E), 2 males, 2 females (NMBAO01016–NMBAO01019).

*Rhampsinitus discolor* (Karsch, 1878): Eastern Cape: Port St. Johns (31.5977°S, 29.5346°E), 2 males (NMBAO01023, NMBAO01024).

*Rhampsinitus leighi* Pocock, 1903: Eastern Cape: Silaka Nature Reserve (31.6529°S, 29.4919°E), 1 male (NMBAO01020); KwaZulu-Natal: Vernon Crookes Nature Reserve (30.27489°S, 30.6092°E), 2 males (NMBAO01021, NMBAO01022).

*Rhampsinitus qachasneki* Kauri, 1962: KwaZulu-Natal: Royal Natal National Park (28.7101°S, 28.9336°E), 1 male, 1 female (NMBAO01025, NMBAO01026).

The specimens were individually hand collected and kept alive until the gonad dissection. Chromosome preparation follows the “plate spreading” method (Traut 1976) widely used in arachnids (e.g. Šťáhlavský and Král 2004). After the dissection, the gonad tissue was hypotonized in 0.075 M KCl for 20 min, subsequently fixed in methanol: acetic acid (3:1) solution for 20 min, and finally dissociated in a drop of 60% acetic acid directly on the surface of the microscope slide. After the dissociation, the slide with the suspension was moved onto a histological plate (45 °C) to allow the evaporation of the liquid component of the suspension. The chromosomes were stained in a 5% Giemsa solution in Sörensen phosphate buffer for 20 min. Chromosomes were photographed using an ORCA-AG monochromatic camera (Hamamatsu) in an Olympus IX81 microscope. The karyotypes were analyzed from photographs using the LEVAN plugin (Sacamato and Zacaro 2009) for IMAGEJ 1.47 program (http://imagej.nih.gov/ij/). We used the classification of chromosomes of Levan et al. (1964) and measured the relative length of the chromosomes for the diploid set (Suppl. material 1).

### FISH detection of 18S rDNA

The probe for 18S rDNA was prepared from *Euscorpius sicanus* (Koch, 1837). The whole genomic DNA was extracted using Genomic DNA Minikit - Tissue (Geneaid), following the manufacturer’s guidelines. The 18S rDNA fragment (GenBank accession number MG761815) were amplified with the following primer combination 18S-Gal forward: 5‘-CGAGCGCTTTTATTAGACCA-3‘ and 18S-Gal reverse: 5‘-GGTTCACCTACGGAAACCTT-3‘ (Fuková et al. 2005), and 50 ng DNA template. The PCR protocol was as follows: 95°C for 3 min, 30 cycles at 94°C for 30 s, 55 °C for 30 s and 72 °C for 2 min, final extension at 72 °C for 3 min. PCR products were purified with GenElute PCR Clean-Up Kit (Sigma-Aldrich). The probe was labelled using PCR with biotin-14-dUTP (Roche) using Nick Translation Kit (Abbott Molecular) following the manufacturer’s guidelines.

The FISH protocol was performed following Forman et al. (2013). Briefly, chromosome preparations were treated with RNase A (200 µg/ml in 2× SSC) for 60 min and then washed twice in 2× SSC for 5 min. Chromosomes were denaturized at 68 °C for 3 min 30 s in 70 % formamide in 2× SSC. The probe mixture, comprising 20 ng of the probe and 2.5 µl of salmon sperm DNA (Sigma-Aldrich) for each slide, was applied. The slides were left to hybridize overnight in a dark chamber at 37 °C. The following day, the preparations were treated with Cy3-conjugated streptavidin, followed by application of biotinylated antistreptavidin and another dose of Cy3-conjugated streptavidin. Chromosome preparations were counterstained with DAPI (4',6-diamidino-2-phenylindole), contained in FluoroshieldTM (Sigma-Aldrich) and observed in an Olympus IX81 microscope equipped with an ORCA-AG monochromatic charge-coupled device camera (Hamamatsu). The images were pseudocolored (red for Cy3 and blue for DAPI) and superimposed with Cell^R software (Olympus Soft Imaging Solutions GmbH).

## Results

### *Guruia africana* (Karsch, 1878)

**Figure 1.**
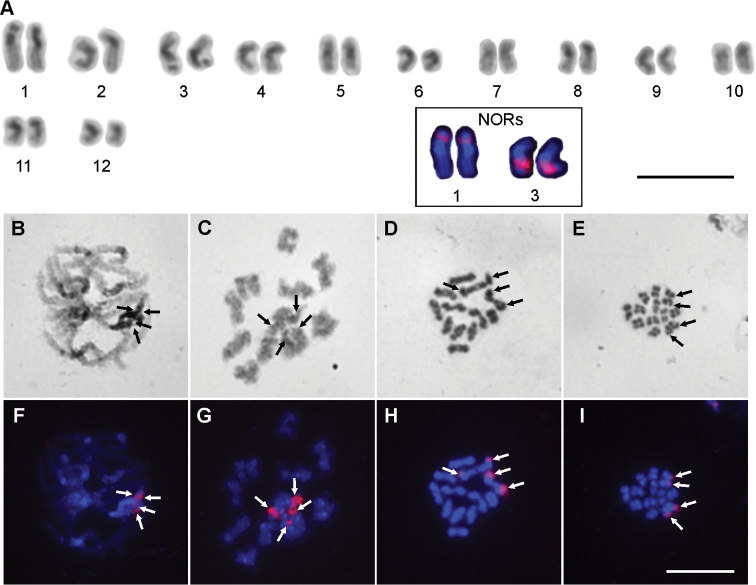
Chromosomes of *Guruia africana* (2n=24) males after Giemsa staining (**A–E**) and after FISH with 18S rDNA (**A** partly, **F–I**). **A** karyotype based on mitotic metaphase **B** pachytene **C** diplotene **D** diakinesis **E** one sister cell of metaphase II **F–I** are the same cells as **B–E** after FISH with 18S rDNA. White arrows indicate the position of 18S rDNA and black arrows indicate the same position after Giemsa staining. Bar = 10 μm.

The diploid number of chromosomes in the four males analysed was 24 (Fig. 1a). The karyotype of this species comprised five pairs of metacentric (pairs No. 1, 3, 5, 6, 9), seven pairs of submetacentric and one pair of subtelocentric (pair No. 12) chromosomes (Suppl. material 1). The chromosomes gradually decreased in length from 6.49 % to 2.74 % of the diploid set (Suppl. material 1). In this species, we observed visible modification of condensation of the chromosomes, such as positive heteropycnosis during mitosis. In the chromosomes, there were visible positive heteropycnotic blocks at the position of the centromere of mitotic metaphases (pairs No. 2, 5, 7, 10) (Fig. 1a); however, in some chromosomes, the heteropycnotic parts expanded to a large proportion of the arms (pairs No. 1, 3, 4, 8, 11), or even across the whole length of the chromosome (pairs No. 6, 9). Large positive heteropycnotic blocks were visible also during prophase I of meiosis, mainly during zygotene and pachytene (Fig. 1b). During the diffuse stage and the diplotene the heteropycnosis was only moderate (Fig. 1c). Later, during diakinesis (Fig. 1d), metaphase I and metaphase II (Fig. 1e), the whole chromosomes seemed to be isopycnotic (Fig. 1d, e). We did not clearly detect visible heteromorphic bivalents during diakinesis and metaphase I (Fig. 1d).

Two pairs of 18S rDNA clusters were detected by FISH in this species. The 18S rDNA probe signals were localized interstitially at approximately one third of the short arm of chromosome pair No. 1 and approximately at one third of the long arm of chromosome pair No. 3 (Fig. 1a). The signals on chromosome pair No. 3 were more intensive than on the longest chromosomes. During pachytene we identified the position of the 18S rDNA clusters on the positive heteropycnotic areas of the chromosomes, and the clusters were usually associated in this phase (Fig. 1f). Also during the diffuse stage and the diplotene the chromosomes bearing the NORs were close to each other (Fig. 1g). The condensation of the chromosomes caused the visualization of the 18S rDNA in the terminal part of the chromosomes during diakinesis and metaphase II (Fig. 1h, i).

### *Rhampsinitus capensis* (Loman, 1898)

**Figure 2.**
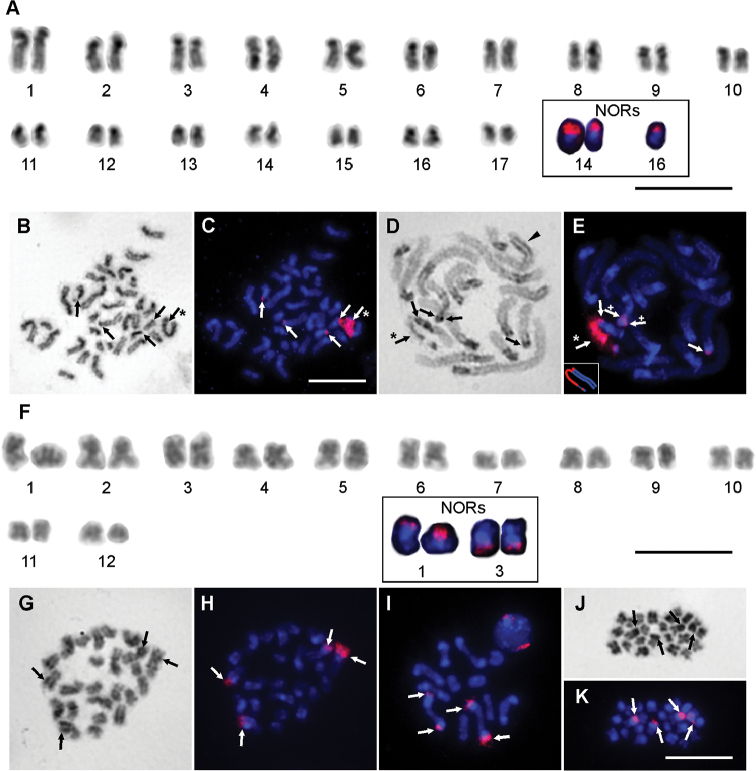
Chromosomes of *Rhampsinitus capensis* (2n=34) (**A-E**) and *Rhampsinitus discolor* (**F–K**) (2n=24) after Giemsa staining (**A, B, D, F, G, J**) and after FISH with 18S rDNA (**A** and **F** partly, **C, E, H, I, K**). **A** male karyotype based on mitotic metaphase **B, C** female mitotic metaphase **D, E** female pachytene, bottom left inset shows a reconstruction of the heteromorphic bivalent; crosses indicate the subterminal position of 18S rDNA; arrowhead indicates bivalent with the expanded positive heteropycnotic area **F** male karyotype based on two sister cells of metaphase II **G, H** male mitotic metaphase **I** diakinesis **J, K** two sister cells of metaphase II. White arrows indicate the position of 18S rDNA and black arrows indicate the position after Giemsa staining. Asterisks indicate large signal of heteromorphic bivalent. Bar = 10 μm.

The diploid number of chromosomes in the two males and two females analysed was 34 (Fig. 2a, b). The karyotype of males comprised two pairs of metacentric (pairs No. 4, 16), three pairs of submetacentric (pairs No. 2, 5, 6, 14), eight pairs of subtelocentric, and four pairs of acrocentric (pairs No. 8, 11, 12, 15) chromosomes (Suppl. material 1). The first pair of chromosomes was slightly longer (5.49 % of the diploid set) than the following chromosome pair (4.50 %), and the rest of the chromosomes gradually decreased in length to 1.49 % of the diploid set (Suppl. material 1). In some chromosomes, positive heteropycnotic blocks were visible at the position of the centromere during mitotic metaphase in both sexes (Figs 2a, b), and also during pachytene in females (Fig. 2d) (we did not observe meiosis in males). During this phase we only observed one pair with the positive heteropycnotic areas expanded to the larger proportion of the arms (Fig. 2d). In pachytene of females we detected one heteromorphic bivalent (Fig. 2d). Three signals of 18S rDNA clusters were detected by FISH in males of this species (Fig. 2a). The 18S rDNA probe signals were localized in the terminal position of the short arms of chromosome pair No. 14 and at one chromosome of pair No. 16. In females, we detected five signals of 18S rDNA clusters. During the mitotic metaphase they seemed to be in a terminal position (Figs 2b, c); however, during the pachytene it was clear that one pair was in a subterminal position (Fig. 2e). Interestingly, one cluster of 18S rDNA was very large (Fig. 2c) and covered nearly the whole arm of the large metacentric chromosome from the heteromorphic bivalent (Fig. 2e).

### *Rhampsinitus discolor* (Karsch, 1878)

The diploid number of chromosomes in the two males analysed was 24 (Fig. 2f). The karyotype comprised eight pairs of metacentric, two pairs of submetacentric (pairs No. 6, 9), and two pairs of subtelocentric (pairs No. 8, 12) chromosomes (Suppl. material 1). The chromosomes gradually decreased in length from 5.96 % to 2.69 % of the diploid set (Suppl. material 1). In this species, we observed modification in spiralization, visible as positive heteropycnosis of large parts of the arms of almost all chromosomes during mitotic prophase. These large blocks of positive heteropycnotic regions were visible only in a few chromosomes later during mitotic metaphase (Fig. 2g). Large positive heteropycnotic blocks were visible also during meiosis, from leptotene. These positive heteropycnotic parts usually associated during pachytene, and they were still clearly visible during diplotene. Some chromosomes still showed the positive heteropycnosis during diakinesis and metaphase II (Fig. 2j). During meiosis, we did not detect clearly visible heteromorphic bivalents (Fig. 2i). Two pairs of 18S rDNA clusters were detected by FISH in this species (Fig. 2h, i, k). The 18S rDNA probe signals were localized at the terminal parts of the first and third pairs of chromosomes (Fig. 2f).

### *Rhampsinitus leighi* Pocock, 1903

**Figure 3.**
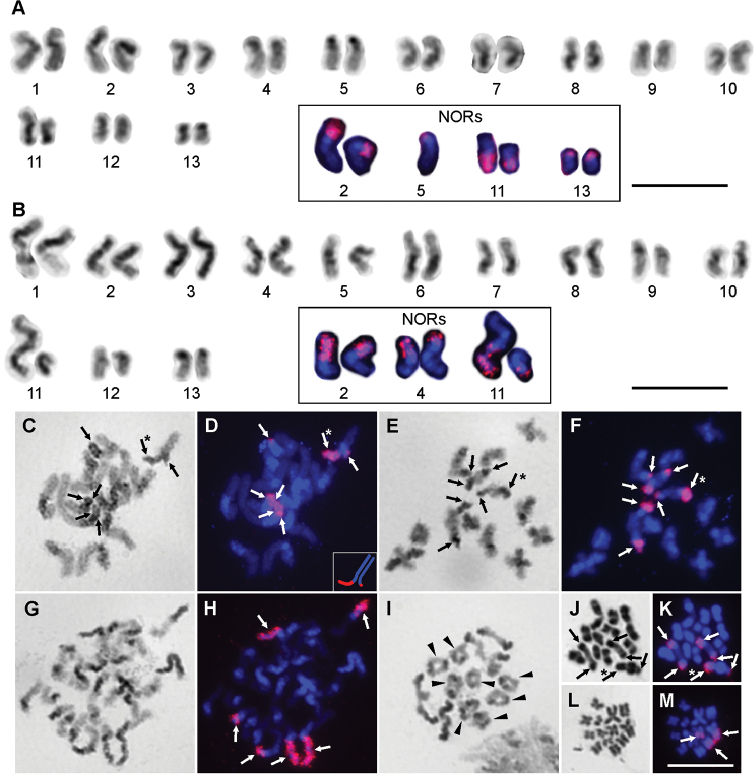
Chromosomes of *Rhampsinitus leighi* (2n=26) from Vernon Crookes (**A, C–F**) and from Silaka (**B, G–M**) after Giemsa staining (**A–C, E, G, I, J, L**) and after FISH with 18S rDNA (**A** and **B** partly, **D, F, H, K, M**). **A, B** karyotypes of males based on mitotic metaphase **C, D** pachytene with heterozygous bivalent (asterisk), bottom right inset shows a reconstruction of the heteromorphic bivalent **E, F** diplotene with heterozygous bivalent (asterisk) **G, H** mitotic prophase **I** diplotene, arrowheads indicate bivalents with two chiasmata. **J, K** diakinesis **L, M** one sister cell of metaphase II. White arrows indicate the position of 18S rDNA and black arrows indicate the same position after Giemsa staining. Asterisks indicate heteromorphic bivalent with large signal of rDNA. Bar = 10 μm.

We identified two cytotypes from two different localities in this species. The diploid numbers of chromosomes in both of them were 26 (Fig. 3a, b), but the morphology of the chromosomes and the number and position of 18S rDNA clusters differed.

The karyotype of cytotype I from Vernon Crookes comprised six pairs of metacentric, five pairs of submetacentric (pairs No. 2, 4, 8, 9, 13), and one pair of subtelocentric (pair No. 5) chromosomes. Moreover, the karyotype of cytotype I included one heteromorphic bivalent (pair No. 11) composed of one metacentric and one submetacentric chromosome. The chromosomes of the first two pairs were distinctly longer (6.18 % and 5.67 % of the diploid set) than the next chromosomes, which gradually decrease in length from 4.29 % to 2.42 % of the diploid set (Suppl. material 1). In some chromosomes, positive heteropycnotic blocks were visible at the position of the centromere in mitotic metaphases, and in some pairs heteropycnotic parts expanded to a large proportion of the arms (Fig. 3a). Large heteropycnotic blocks were also visible during the early meiotic phases such as zygotene, pachytene (Fig. 3c) and diplotene (Fig. 3e). Later, during metaphase I and metaphase II the whole chromosome seemed to be isopycnotic. We only found bivalents with one chiasma in both males from Vernon Crookes during the diplotene (Fig. 3e, Suppl. material 2). Altogether seven signals of 18S rDNA clusters were detected by FISH in cytotype I (Fig. 3f). All of them were localized in the terminal position of the chromosomes. Interestingly, we identified a heterozygous state in two pairs of chromosomes (Fig. 3a). We identified a small signal in the terminal position of one chromosome of pair No. 5 (Fig. 3a), which is also visible during pachytene (Fig. 3d) and diplotene (Fig. 3f). In pair No. 11 we identified an expanded part of one chromosome that covered 18S rDNA clusters, which makes the bivalent heteromorphic (compare Fig. 3c, d, e, f).

The karyotype of cytotype II from Silaka (Fig. 3b) comprised seven pairs of metacentric and five pairs of submetacentric (pairs No. 3, 7, 9, 10, 12) chromosomes. Similar to cytotype I, cytotype II also included one heteromorphic bivalent (pair No. 11) composed of one metacentric and one submetacentric chromosome (Suppl. material 1). The chromosomes of the first three pairs were slightly longer (6.04 %, 5.66 %, and 5.10 % of the diploid set) and the following chromosomes gradually decreased in length from 4.42 % to 2.21 % of the diploid set (Suppl. material 1). Also in cytotype II, positive heteropycnotic blocks were visible at the position of the centromere of the chromosomes during mitotic prometaphase (Fig. 3g) and metaphase, and in some pairs the heteropycnotic parts expanded to a large portion of the arms (Fig. 3b). Large heteropycnotic blocks were also visible during the early meiotic phases until diplotene (Fig. 3i). Later, during diakinesis, metaphase I and metaphase II, the whole chromosome seemed to be isopycnotic (Figs 3j, l). We found from four to eight bivalents with two chiasmata in the male from Silaka during diplotene (Fig. 3i, Suppl. material 2). Six signals of 18S rDNA clusters were detected by FISH in cytotype II (Figs 3b, h, k). All of them were localized in the terminal position of the chromosomes (Figs 3b, h, k, m). We detected a heterozygous state in one pair of chromosomes (Fig. 3b). In pair No. 11 we identified an expanded part of one chromosome that covered 18S rDNA clusters, which makes the bivalent heteromorphic (compare Fig. 3j, k).

### *Rhampsinitus qachasneki* Kauri, 1962

**Figure 4.**
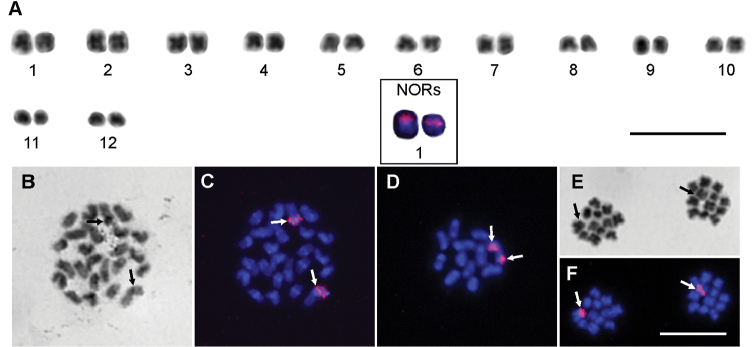
Chromosomes of *Rhampsinitus qachasneki* (2n=24) males after Giemsa staining (**A, B, E**) and after FISH with 18S rDNA (**A** partly, **C, D, F**). **A** male karyotype based on two sister cells of metaphase II **B, C** mitotic metaphase **D** diakinesis **E, F** two sister cells of metaphase II. White arrows indicate the position of 18S rDNA and black arrows indicate the same position after Giemsa staining. Bar = 10 μm.

The diploid number of chromosomes in this species was 24 (Fig. 4a, b). The karyotype of the male comprised six pairs of metacentric, four pairs of submetacentric (pair No. 2, 6, 9, 11), and two pairs of subtelocentric (pair No. 8, 12) chromosomes (Suppl. material 1). The chromosomes gradually decreased in length from 5.74 % to 2.87 % of the diploid set (Suppl. material 1). In some chromosomes, positive heteropycnotic blocks were visible at the position of the centromere of mitotic metaphases, but these blocks did not expand across the whole arms of the chromosomes. Large heteropycnotic blocks were also visible during the early phases of meiosis such as zygotene, pachytene and diplotene; however, later during the metaphase I and metaphase II the entire chromosomes seemed to be isopycnotic (Fig. 4e). One pair of 18S rDNA clusters was detected by FISH in the terminal part of one pair of chromosomes in this species (pair No. 1) (Fig. 4a, c, d, f).

## Discussion

Phalangiid harvestmen have a centre of diversity in the Northern Hemisphere (Giribet and Kury 2007), and their presence in South Africa is traditionally explained by dispersal from the Mediterranean region (Staręga 1984). In this study, we focus on the genera *Guruia* and *Rhampsinitus* that are endemic to the Afrotropical biogeographical region (Staręga 1984), in order to provide further information about the cytogenetics of the family Phalangiidae from previously understudied parts of the World.

The results of the cytogenetic analyses of *Guruia africana* (2n=24) and four species of the genus *Rhampsinitus* (2n=24, 26, 34) show that the African taxa share the basic karyotype characteristics with the Northern Hemisphere phalangiids analysed so far (13 species). However, detailed information about the karyotypes is only available in three species, whereas the records for the remaining species only comprise chromosome numbers (see Tsurusaki et al. 2017). In Phalangiidae the chromosome number ranges from 2n=16 in *Oligolophus tridens* (C.L. Koch, 1836) (Tsurusaki 2007) to 2n=36 in *Rilaena triangularis* (Herbst, 1799) (Sokolow 1930), with the karyotypes predominated by biarmed chromosomes. The most frequent number of chromosomes in this group is 2n=24 (8 species) and 2n=32 (5 species) (Tsurusaki et al. 2017, present study). The hypothesised ancestral number of chromosomes in the order Opiliones is around 30 (Svojanovská et al. 2016). The low numbers of the chromosomes in Eupnoi may be explained in two different ways. The first hypothesis assumes independent reductions of the proposed ancestral number of chromosomes shared by the whole order in each family of Phalangioidea. The family Phalangiidae still harbours some species with an ancestral number close to 30 (see Tsurusaki et al. 2017). Therefore the 2n=34, found in *Rhampsinitus capensis* (the highest number of chromosomes), would represent a karyotype derived by centric fissions, since one-armed chromosomes (subtelocentrics and acrocentrics) are still common in this species’ karyotype. The rest of *Rhampsinitus* species (2n=24 and 26) and *Guruia africana* (2n=24) would thus possess karyotypes derived by chromosome rearrangements, like fusions and inversions. This hypothesis would imply an independent reduction of the diploid number in the families Protolophidae (2n=18–22) and Sclerosomatidae (2n=10–24) (see Tsurusaki et al. 2017). The diploid number 2n=30 is also known from the family Caddidae (Tsurusaki and Cokendolpher 1990), a sister group to the superfamily Phalangioidea (Giribet et al. 2010), which would further indicate that the diploid number close to 30 probably represents the ancestral state in Eupnoi.

Alternatively, the second hypothesis presumes the ancestral number of chromosomes of the whole suborder Eupnoi being between 20 and 24 (the most frequent number in this suborder; see Tsurusaki et al. 2017). In this case, all higher numbers of chromosomes would represent a derived state driven by chromosomal fissions. However, the direction of karyotype evolution in Eupnoi can only be resolved by interpreting the cytogenetic data within a wider phylogenetic framework. Unfortunately, a robust phylogenetic scheme for Eupnoi is not currently available.

The data concerning the number and position of the 18S rDNA clusters using the FISH technique in arachnids are still limited (see Svojanovská et al. 2016), and most of them come from scorpions (e.g. Mattos et al. 2014, Sadílek et al. 2015). The results suggest that the position of 18S rDNA clusters can be highly conserved among different genera/species of scorpions, e.g. in a terminal position of the genera *Rhopalurus* Thorell, 1876 and *Tityus* C. L. Koch, 1836 (e.g. Mattos et al. 2014) from South America, or in an interstitial position in *Androctonus* Ehrenberg, 1828 from Africa and Asia (Sadílek et al. 2015).

Contrastingly, the variability of the number of 18S rDNA sites may be high in some arachnids (e.g. Forman et al. 2013). However, the interpretation of the differences in the number of 18S rDNA clusters requires a precise knowledge of both the ancestral number of these clusters and the phylogeny of the group, otherwise the course of the karyotype evolution cannot be specified (e.g. Svojanovská et al. 2016), as in case of our results. There is no information available directly concerning the 18S rDNA sites of phalangiid harvestmen in the literature that would allow a comparison to our results. The only record available for the entire suborder Eupnoi belongs to the sclerosomatid harvestman *Psathyropus tenuipes* (L. Koch, 1878), which possesses one NOR (a transcription unit that encodes 28S, 5.8S and 18S rRNAs) in the diploid set (Gorlov and Tsurusaki 2000b). However, the NOR was visualised by silver staining, which only detects the NORs that are active during the preceding interphase (Miller et al. 1976), and thus the real number may be underestimated (Forman et al. 2013). Generally, it is hypothesized that one pair of NORs, as well as 18S rDNA sites, represents the ancestral state in the class Arachnida (Forman et al. 2013). We detected one pair of 18S rDNA clusters only in one species included in the analyses (*Rhampsinitus qachasneki*), while the number of clusters varied between 2 in *R. qachasneki* and 7 in *R. leighi*. The high variability in the number, position and even size of rDNA clusters between and also within the analysed species suggests intensive chromosomal changes and rapid evolutionary dynamics of 18S rDNA clusters on chromosomes of South African phalangiids. *Rhampsinitus* has a terminal position of 18S rDNA clusters, similarly to basal harvestmen of the suborder Cyphophthalmi (Svojanovská et al. 2016), and other arachnid groups such as spiders (e.g. Král et al. 2013), amblypygids (Paula-Neto et al. 2013), and South American scorpions (e.g. Mattos et al. 2014). The proximity to the telomeric region facilitates the efficiency of ectopic recombination (Goldman and Lichten 1996) and the preferential replacement of rDNA genes into a new subtelomeric position (Nguyen et al. 2010), as detected in all *Rhampsinitus* species. The effect of ectopic recombination may also be increased due to the association of positive heteropycnotic areas of different chromosomes during the meiotic prophase (e.g. Figs 1b, 3c). However, *Guruia africana* has 18S rDNA clusters placed in an interstitial position of one arm of biarmed chromosomes, which may be a consequence of another chromosome rearrangements, transposable element insertions or ectopic recombination (see Cabrero and Camacho 2008). Some 18S rDNA clusters in *Rhampsinitus* species were longer (see Fig. 3a, b), likely due to the presence of a higher number of copies of the 18S rDNA elements. This observation can be also explained by the duplication of the chromosome part including this cluster, or alternatively as a consequence of an insertion of transposable elements, which potentially accelerates the genomic reorganization after a speciation event (e.g. Symonová et al. 2013).

We also detected intraspecific variability and a heterozygous state in *R. capensis* and *R. leighi* that may correspond to population polymorphism. The low dispersal capability, typical for most harvestmen (Giribet and Kury 2007), can lead to a genetic differentiation among the populations on a relatively small geographical scale in many arachnid groups (e.g. Kotrbová et al. 2016, Opatova et al. 2016). The intraspecific variability in the number of 18S rDNA clusters between the populations from the two localities of *R. leighi* could thus be explained as an independent accumulation of the chromosomal changes at each locality, as is assumed for the wolf spider *Wadicosa fidelis* (O. Pickard-Cambridge, 1872) (Forman et al. 2013). However, the harvestmen are also an understudied group, so the differences in 18S rDNA clusters and karyotypes between both populations could indicate a possible existence of cryptic species that are common in this order (e.g. Murienne et al. 2010). Given this scenario, the presence of a heterozygous state could be a result of crossbreeding.

Interestingly, we also identified heteromorphic bivalents in both populations of *R. leighi* caused by distinctly enlarged 18S rDNA clusters on one chromosome. Similarly, the heteromorphic bivalents were also detected in *R. capensis*, but the enlarged rDNA sites were only identified in females. The heterozygous states in the size of 18S rDNA clusters could indicate the existence of XY sex chromosomes in *R. leighi* and ZW sex chromosomes in *R. capensis*. The XY sex chromosome system is known in the harvestmen *Sabacon makinoi* Suzuki, 1949 (Dyspnoi, Sabaconidae) (Tsurusaki 1989) and from 13 species of Sclerosomatidae (Eupnoi) (see Tsurusaki et al. 2017). In other arachnid orders the XY system is common in ticks (Oliver 1977), but it was also identified in few species from different spider families (Král et al. 2006, Řezáč et al. 2006), and some species of the pseudoscorpion family Neobisiidae (Troiano 1990, 1997, Šťáhlavský and Zaragoza 2008). The ZW system is completely exceptional in arachnids (Tsurusaki 2007). It is described only from *Mitopus morio* and it is hypothesized in *Olioglophus aspersus* (Karsch, 1881) (both Phalangiidae) (Tsurusaki and Cokendolpher 1990). However, the ZW system in *M. morio* was described from a low quality single standard stained mitotic metaphase of one female (see Tsurusaki and Cokendolpher 1990). In this study, heteromorphic bivalents were detected in both females of *R. capensis* (Fig. 2b, d); however, we did not obtain any information about meiosis and chromosome pairing in males. The number and position of 18S rDNA clusters in mitotic metaphases of the analysed males does not indicate the presence of homomorphic chromosomes that would correspond to the heteromorphic state in females. Similarly, the evidence of a XY system in *R. leighi* is unclear, since we have no information concerning the organization of 18S rDNA clusters in females.

The size variation of rDNA clusters on homologous chromosomes is frequently considered a result of unequal crossing-over or gene duplication that does not represent sex-specific differences (e.g. Foresti et al. 1981). However, when the sex locus is located close to the rDNA, the recombination in this region can be limited and thus accelerate the evolution of the sex chromosome(s) (Reed and Phillips 1997). For that reason, we cannot rule out that the heteromorphic bivalents in *R. leighi* and *R. capensis* may represent the initial stages of sex differentiation from the ancestral nondifferentiated state typical for harvestmen (Tsurusaki 2007). Our analysis indicates that the differences in morphology and size of the chromosomes, considered in some harvestmen to be the sex chromosomes (e.g. Tsurusaki and Cokendolpher 1990), may in fact represent the variability in rDNA clusters induced by ectopic recombination, unequal crossing-over or gene duplication mentioned above. It would be necessary to analyse the species with heteromorphic sex chromosomes in order to know whether this heteromorphism is also associated with the rDNA. These findings bring a new light to our understanding of the presence of heteromorphic bivalents in harvestmen, and they highlight the necessity for the implementation of molecular cytogenetic techniques such as CGH in order to assess the presence of sex chromosomes in this group.

## Supplementary materials

Supplementary material 1 Table S1 Data type: Microsoft Excel Worksheet (.xls). Explanation note: Measurements of the relative chromosome length (% RCL) and arm ratio of South African harvestmen from family Phalangiidae (± standard deviation). Numbers of measured metaphases are given in brackets following the species names. This is an open access article distributed under the terms of the Creative Commons Attribution License (CC BY 4.0), which permits unrestricted use, distribution, and reproduction in any medium, provided the original author and source are credited.František Šťáhlavský, Vera Opatova, Pavel Just, Leon N. Lotz, Charles R. Haddad

Supplementary material 2 Table S2 Data type: Microsoft Excel Worksheet (.xls). Explanation note: Numbers of nuclei with different numbers of chiasmata in diplotene of *Rhampsinitus leighi* (2n = 26) from Vernon Crookes (cytotype I) and Silaka (cytotype II). This is an open access article distributed under the terms of the Creative Commons Attribution License (CC BY 4.0), which permits unrestricted use, distribution, and reproduction in any medium, provided the original author and source are credited.František Šťáhlavský, Vera Opatova, Pavel Just, Leon N. Lotz, Charles R. Haddad
